# A new species of *Mengenilla* (Insecta, Strepsiptera) from Tunisia

**DOI:** 10.3897/zookeys.198.2334

**Published:** 2012-05-30

**Authors:** Hans Pohl, Oliver Niehuis, Kai Gloyna, Bernhard Misof, Rolf G. Beutel

**Affiliations:** 1 Entomology Group, Institut für Spezielle Zoologie and Evolutionsbiologie, Friedrich-Schiller-University Jena, Erbertstraße 1, 07743 Jena, Germany; 2 Zentrum für Molekulare Biodiversitätsforschung, Zoologisches Forschungsmuseum Alexander Koenig, Adenauerallee 160, 53113 Bonn, Germany; 3 TL Bio-Test Labor GmbH Sagerheide, Birkenallee 19, 18184 Sagerheide, Germany

**Keywords:** Mengenillidae, *Mengenilla*, new species, taxonomy

## Abstract

A new species of *Mengenilla* Hofeneder, 1910 (Strepsiptera, Mengenillidae) from southern Tunisia is described. *Mengenilla moldrzyki* **sp. n.** can be distinguished from congeners by a slightly emarginated posterodorsal margin of the head, compound eyes with a light tan dorsal part, mandibles with a narrow distal part, and a v-shaped pronotum. With the description of *Mengenilla moldrzyki* **sp. n.**, eleven valid species of *Mengenilla* are currently recognised. *Mengenilla moldrzyki* **sp. n.** is the third species of the genus with known females and female puparia. First instar larvae, endoparasitic larval stages, the male puparium and the host are unknown. The new species is also the first strepsipteran with a fully sequenced genome.

## Introduction

Mengenillidae is a basal group of Strepsiptera. The recently described *Bahiaxenos relictus* Bravo, Pohl, Silva-Neto and Beutel, 2009 (Bahiaxenidae) from Brazil is the sistergroup of all other strepsipteran families, and Mengenillidae represents the second branch within the order, i.e. the sistergroup of all families with pterygote hosts (Stylopidia) (Pohl and Beutel 2005, Bravo et al. 2009). Plesiomorphic features including free-living females, comparatively broad mandibular bases, and the absence of specialized hairy soles on the tarsomeres of males characterize the group. Recently, the monophyly of Mengenillidae was challenged. If only characters of males are analysed the mengenillid genera *Eoxenos*, *Congoxenos*, and *Mengenilla* split successively from the strepsipteran phylogenetic backbone after *Bahiaxenos* (Pohl and Beutel 2005, Bravo et al. 2009, Hünefeld et al. 2011). The clarification of this issue will require detailed morphological information on males, females and larvae – information that is presently still very fragmentary. The monophyly of *Mengenilla* is well established and supported by the following apomorphies of males: reduced primary and secondary mandibular joint, immobilized maxilla, and a completely undivided labium (Pohl and Beutel 2005). Further diagnostic features of the males are six-segmented antennae, flabella on antennomeres 3–5, five-segmented tarsi with well developed claws, a prominent and elongated abdominal segment X, and a nearly straight penis with a pointed apex (Kinzelbach 1970, 1978, Cook 2007).

**Figure 1.**
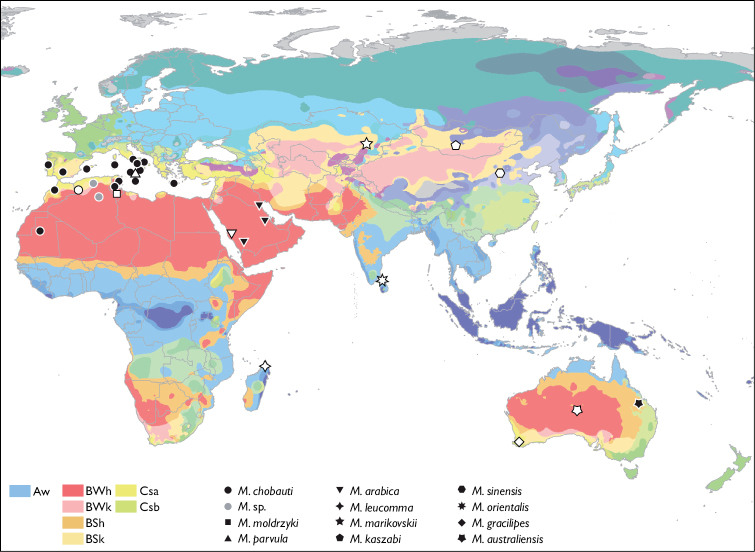
Distribution of the genus *Mengenilla* plotted on the Köppen-Geiger climate type map (map modified after Peel et al. (2007)). Type localities with open symbols. Grey dots: *Mengenilla* sp. mentioned by Hofeneder (1928) and de Peyerimhoff (1919). Description of the climate symbols (only where *Mengenilla* occurs): **Aw** Tropical, savannah; **BWh** Arid, desert, hot; **BWk** Arid, desert, cold; **BSh** Arid, steppe, hot; **BSk** Arid, steppe, cold; **Csa** Temperate, dry summer, hot summer; **Csb** Temperate, dry summer, warm summer. For a detailed explanation of the map and symbols see Peel et al. (2007).

A total of 19 species have been described in the genus *Mengenilla*, 10 of which are currently recognised as valid (Cook 2007). The genus is restricted to the Old World and occurs in xerotherm, semi-arid and arid areas, with the exception of *Mengenilla orientalis* Kifune and Hirashima, 1980 from Sri Lanka and *Mengenilla leucomma* Cook, 2007 from Madagascar (Kinzelbach 1979, Kifune and Hirashima 1980, Cook, 2007, Bravo et al. 2009). *Mengenilla chobauti* Hofeneder, 1910 and *Mengenilla parvula* Silvestri, 1941 occur in the Mediterranean region. While *Mengenilla parvula* is restricted to Sicily, *Mengenilla chobauti* is recorded from North Africa, Spain, Portugal, Crete, Malta, Italy, including Sicily and Sardinia and has the widest distribution of all described *Mengenilla* species (Silvestri 1941, 1943, Kinzelbach 1978). Nearly all other species are only known from their type locality. Figure 1 gives an overview of the described species of the genus and their distribution. Males are known for all currently described species of *Mengenilla*, but females, immature stages and hosts only for *Mengenilla chobauti* and *Mengenilla parvula*. The presently known hosts are lepismatid Zygentoma: *Ctenolepisma ciliata* (Dufour, 1831) for *Mengenilla chobauti*, and *Ctenolepisma michaelseni* Escherich, 1905 for *Mengenilla parvula*, respectively (Silvestri 1943).

In this contribution, we describe a new species of *Mengenilla* from southern Tunisia. In October 1999, the first author (HP) collected more than 260 male specimens at a light trap in the Tunisian Sahara. Despite an intensive search, females were neither found by that time nor during a second collecting trip in 2010. In 2005, Uwe Moldrzyk (Berlin) collected a single female within its puparium. Several studies have already been published on males of this species without a formal description (referred to as “*Mengenilla* sp., undescribed species Tunisia”). They cover the head morphology (Beutel and Pohl 2006), effects of miniaturization (Beutel et al. 2005), the male postabdomen, and genital structures (Hünefeld and Beutel 2005, Hünefeld et al. 2011). Finally, the complete genome of this species has been sequenced, which is also the first published complete genome of a twisted-wing parasite (Niehuis et al. in press). *Mengenilla moldrzyki* sp. n. is consequently the best investigated strepsipteran species thus far.

## Methods

The morphological terminology for the head is based on Beutel and Pohl (2006) and for the thorax and abdomen on Kinzelbach (1971, 1978), except for the term “aede- agus”, which is replaced by the term “penis” as suggested by Hünefeld et al. (2011). Species descriptions are based on a designated holotype but all available specimens were taken into account in order to assess the intraspecific variation.

Photographs of critical point dried specimens were taken with a Nikon D 90 digital SLR equipped with a 40 mm and with a 63 mm Zeiss Luminar macro lense, plus an adjustable extension bellows. The specimens were illuminated by two flashlights fitted with a transparent cylinder for even and soft light. Helicon Focus Mac Pro X64 was used to combine a stack of several partially focused images. For scanning electron microscopy images (SEM) and macro photography, specimens were dehydrated using increasing steps of ethanol up to 100% and dried at the critical point (Emitech K850 critical point dryer). For SEM, specimens were subsequently sputter-coated (Emitech K500) and examined on a Philips XL30 ESEM using a rotatable specimen holder (Pohl 2010).

To assess intraspecific variation, 74 male specimens of *Mengenilla moldrzyki* sp. n. were prepared on slides and embedded in Euparal (Chroma, Münster, Germany). To avoid shrink-artefacts, specimens were dehydrated using increased steps of ethanol up to 96% prior to preparation, followed by several steps of Euparal Essenz in increasing concentration (diluted with 96% ethanol) up to 80% and then finally embedded in Euparal. An antenna, a maxilla, legs, the hind wing of one side, and the penis were prepared and each covered separately with a coverslip. Measurements of the 74 prepared males were performed using an Olympus SZ 40 stereomicroscope and an Olympus BX 50 microscope with a calibrated ocular micrometer. Measurements of the female and of the female puparium were performed using SEM micrographs and macro photographs. Definitions of the measurements are illustrated in figure 4 and in figure 5. Morphological features were illustrated using a 10×10 ocular grid on either an Olympus SZ 40 stereo microscope or an Olympus BX 50 microscope. Drawings of features of the females are based on SEM micrographs. The colouration of different body parts of the specimens are specified after the List of colours (Wikipedia).

To corroborate our assumption that the males and the females described here indeed belong to the same species, i.e., *Mengenilla moldrzyki* sp. n., we compared a 741-bp long section of their mitochondrial gene COI – an established barcoding gene. DNA was extracted from a small part of the abdomen of the adult female using the QIAGEN DNeasy Blood & Tissue Kit and following the protocol for insects (QIAGEN GmbH, Hilden, Germany). The COI marker region was PCR-amplified with the oligonucleotide primers 5’-TAG GGG TTA GAT CAG GTT GA-3’ and 5’-AGG ACA TAG TGG AAA TGT GC-3’. The oligonucleotide primers had been specifically designed for this purpose with the software Primer3 (Rozen and Skaletsky 2000), using the COI sequence from the *Mengenilla moldrzyki* genome project (Niehuis et al. in press) as reference and template. Note that the *Mengenilla moldrzyki* genome has been sequenced using the DNA from males (Niehuis et al. in press). The PCR was conducted in a 20 μl volume consisting of 0.5x QIAGEN Q-Solution, 1x QIAGEN Multiplex PCR Master Mix (QIAGEN GmbH, Hilden, Germany), 0.8 μM of each oligonucleotide primer, and ~ 50 ng DNA. The PCR temperature profile started with an initial denaturation and QIAGEN HotStarTaq DNA polymerase activation step at 95° C for 15 min., followed by 35 cycles of 95° C for 1 min., 49° C for 1 min., and 72° C for 1 min, followed by 10 min. at 72° C. The PCR product was purified with the QIAquick PCR Purification Kit (QIAGEN GmbH, Hilden, Germany) and send to Macrogen Inc. (Amsterdam, Netherlands) for direct sequencing with the above oligonucleotide primers. Forward and reverse DNA strands were assembled to contigs, trimmed (to exclude the binding sites of the oligonucleotide primers), and aligned to the COI reference sequence from the *Mengenilla moldrzyki* genome project (Niehuis et al. in press) with GENEIOUS PRO 5.5.3 (Drummond et al. 2010). The assembled COI sequence has been deposited in the European Nucleotide Archive (ENA) und is available under the accession number HE610110.

The information for the specimens is given in a standard manner, i.e., locality, geographic coordinates, elevation, date of collection (month indicated in lower case Roman numerals), habitat information, collector, depository, and preparation. Male (♂) and female (♀) symbols indicate the sex.

The specimens referred to below along with the abbreviations used in the text are deposited in the following collections: NHM – Natural History Museum, London, Great Britain; NMNH – Smithsonian Institution National Museum of Natural History, Washington, USA; HP – Private collection Hans Pohl, Jena, Germany; PMJ – Phyletisches Museum, Jena, Germany; SDEI – Senckenberg Deutsches Entomologisches Institut, Müncheberg, Germany.

## Taxonomy

### Mengenilla moldrzyki sp. n.

urn:lsid:zoobank.org:act:131705B7-8BD1-4983-A8B3-84D4D26284D1

http://species-id.net/wiki/Mengenilla_moldrzyki

#### Etymology.

The species is named after the collector of the female, Uwe Moldrzyk (Berlin).

#### Diagnosis.

Six-segmented antennae with flabella on antennomeres 3–5, immobilized maxillae, a completely undivided labium, and five-segmented tarsi with well developed claws are generic features of the genus *Mengenilla* verified in the new species.

The males are distinguished from congeners as follows (*Mengenilla marikovskii* Medvedev, 1970 from south-eastern Kazakhstan is excluded from the key, because the description and illustrations are too superficial. In contrast to the statement of Medvedev (1970), the type series of that species is not deposited in the Zoological Institute, Russian Academy of Sciences, St. Petersburg, Russia and could not be re-examined.):

**Table:** 

1	Antenna relatively short (less than 2 times the head length)	2
–	Antenna relatively long (more than 2 times the head length)	4
2	Dorsomedian frontal impression of head absent, with narrow free labrum, Australia	*Mengenilla australiensis* Kifune & Hirashima, 1983
–	Head with dorsomedian frontal impression, labrum absent	3
3	Metapostscutellum long (~0.4 times the metanotal length), ~50 ommatidia, maxillary palp longer than proximal part of maxilla, Sri Lanka	*Mengenilla orientalis* Kifune & Hirashima, 1980
–	Metapostscutellum comparatively short (~0.3 times the metanotal length), ~60 ommatidia, maxillary palp subequal to proximal part of maxilla, China (Shansi)	*Mengenilla sinensis* Miyamoto, 1960
4	25–27 ommatidia, with small free labrum, Australia	*Mengenilla gracilipes* (Lea, 1910)
–	>30 ommatidia, labrum absent	5
5	Mandible with broad distal part (compare Fig. 3B)	6
–	Mandible with narrow distal part (compare Fig. 3A)	7
6	35–38 ommatidia, proximal part of maxilla globular, maxillary palp attached apically, body length 1.7–3.1 mm, Italy (Sicily)	*Mengenilla parvula* (Silvestri, 1941)
–	35–90 ommatidia, proximal part of maxilla slender, maxillary palp attached subterminally, body length 2.6–5.9 mm, Algeria, Morocco, Tunisia, Italy (including Sardinia and Sicily), Spain, Portugal	*Mengenilla chobauti* Hofeneder, 1910
7	Compound eye with uniform colouration, maxillary palp attached subterminally	8
–	Dorsal part of compound eye white to light tan, maxillary palp attached apically	9
8	Tips of flabella and 6^th^ antennomere rounded, 37–39 ommatidia, body length 2.8–3.1 mm, Saudi Arabia	*Mengenilla arabica* Kinzelbach, 1979
–	Tips of flabella and 6^th^ antennomere acuminate, 45–65 ommatidia, maxilla with conspicuous extension, body length 3.2–5.1 mm, Mongolia	*Mengenilla kaszabi* Kinzelbach, 1970
9	Posterodorsal margin of head deeply concave, pronotum rectangular, anterior margin slightly emarginated, colour of thorax light brown, body length 2.4–2.8 mm, Madagascar	*Mengenilla leucomma* Cook, 2007
–	Posterodorsal margin of head slightly emarginated, pronotum v-shaped, anterior margin concave, colour of thorax dark brown, body length 3.4–4.8 mm, Tunisia	*Mengenilla moldrzyki* sp. n.

The female of *Mengenilla moldrzyki* sp. n. is distinguished from *Mengenilla chobauti* and *Mengenilla parvula* by the much more slender distal part of its mandible,and from *Mengenilla parvula* additionally by its longer scapus. The female puparium is distinguished from that of *Mengenilla chobauti* and *Mengenilla parvula* by the complete absence of cuticular thorns, a rounded anterior prothoracic margin with rounded anterolateral edges, and a tapering caudal margin of the abdomen (Figs 12, 13).

#### Description of the male (Figs 2–6).

Measurements (male holotype, followed by minimum, maximum of paratypes, and mean values of all measured specimens in parentheses, critical point dried specimens and specimens in ethanol not measured, in µm): 1. total length 4,000 (3,450–4,785, avg. 4,182), 2. width of head 788 (613–875, avg. 762), 3. length of head 300 (250–350, avg. 296), 4. width between compound eyes 225 (188–300, avg. 237), 5. number of ommatidia (average of three counts) 71±2 (44–77, avg. 65), 6. total length of antenna 1,015 (821–1,075, avg. 963), 7. length of flabellum of 3^rd^ antennomere 940 (710–990, avg. 864), 8. length of flabellum of 4^th^ antennomere 900 (700–960, avg. 845), 9. length of flabellum of 5^th^ antennomere 830 (610–890, avg. 785), 10. length of 6^th^ antennomere 720 (580–790, avg. 695), 11. length of mandible 360 (275–375, avg. 331), 12. width of mandible 125 (105–145, avg. 122), 13. total length of maxilla including palp 260 (185–345, avg. 256), 14. length of proximal part of maxilla (cardo+stipes) 110 (65–125, avg. 89), 15. length of maxillary palp 200 (150–270, avg. 207), 16. length of pronotum 188 (125–213, avg. 167), 17. width of pronotum 463 (413–588, avg. 479), 18. width of mesonotum 525 (463–663, avg. 542), 19. length of metanotum 1,910 (1,540–2,150, avg. 1,862), 20. width of metathorax 1050 (750–1,150, avg. 971), 21. length of postlumbium 363 (275–488, avg. 373), 22. length of metapostscutellum 600 (413–688, avg. 550), 23. length of hind wing 3,440 (2,820–3,800, avg. 3,424), 24. length of procoxa 540 (440–590, avg. 525), 25. length of prothrochanterofemur 620 (500–680, avg. 600), 26. length of protibia 520 (410–600, avg. 506), 27–31. length of protarsi (proximal to distal) 310, 170, 135, 100, 180 (240– 370, avg. 314) (135–205, avg. 177) (90–195, avg. 144) (80–140, avg. 109) (140–220, avg. 175), 32. length of mesocoxa 670 (520–720, avg. 624), 33. length of mesothrochanterofemur 680 (550–760, avg. 664), 34. length of mesotibia 530 (410–630, avg. 522), 35–39. length of mesotarsi (proximal to distal) 315, 160, 130, 100, 185 (260–380, avg. 324) (140–210, avg. 176) (105–195, avg. 146) (80–140, avg. 109) (145–220, avg. 181), 40. length of metatrochanter 190 (170–255, avg. 216), 41. length of metafemur 570 (450–630, avg. 548), 42. length of metatibia 500 (380–580, avg. 480), 43–47. length of metatarsi (proximal to distal) 280, 150, 135, 95, 180 (215–345, avg. 290) (90–195, avg. 164) (105–185, avg. 141) (75–130, avg. 102) (145–220, avg. 180), 48. length of penis 355 (285–390, avg. 346).

**Figure 2.**
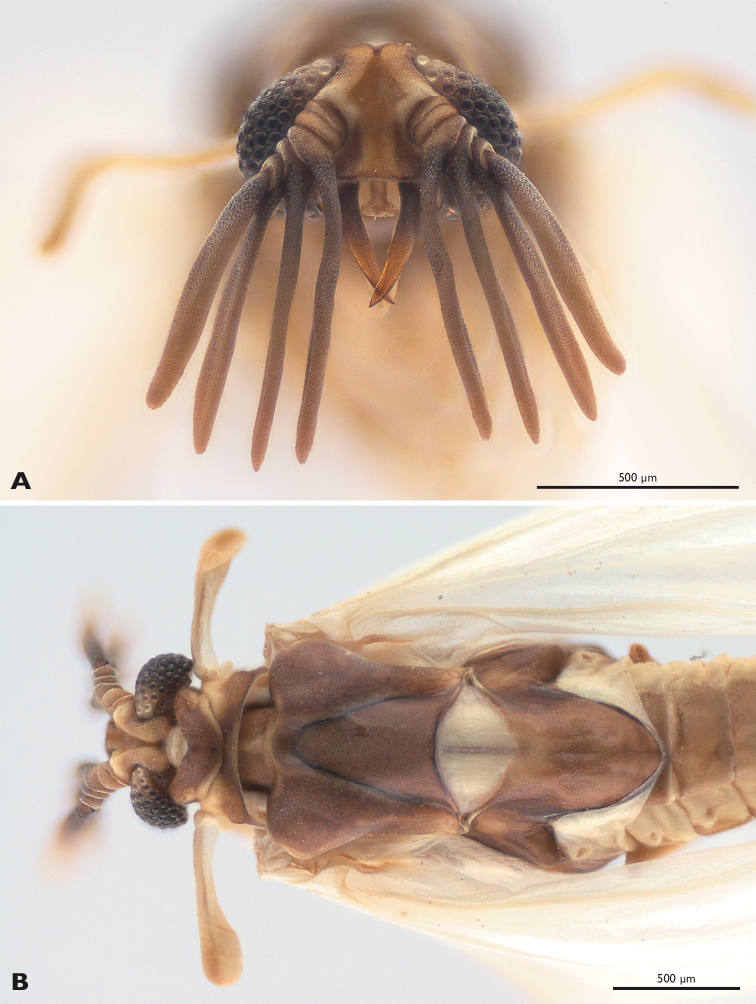
*Mengenilla moldrzyki* sp. n. ♂ **A** Head frontal view **B** Head, thorax and anterior part of abdomen, dorsal view; photomicrograph.

**Figure 3.**
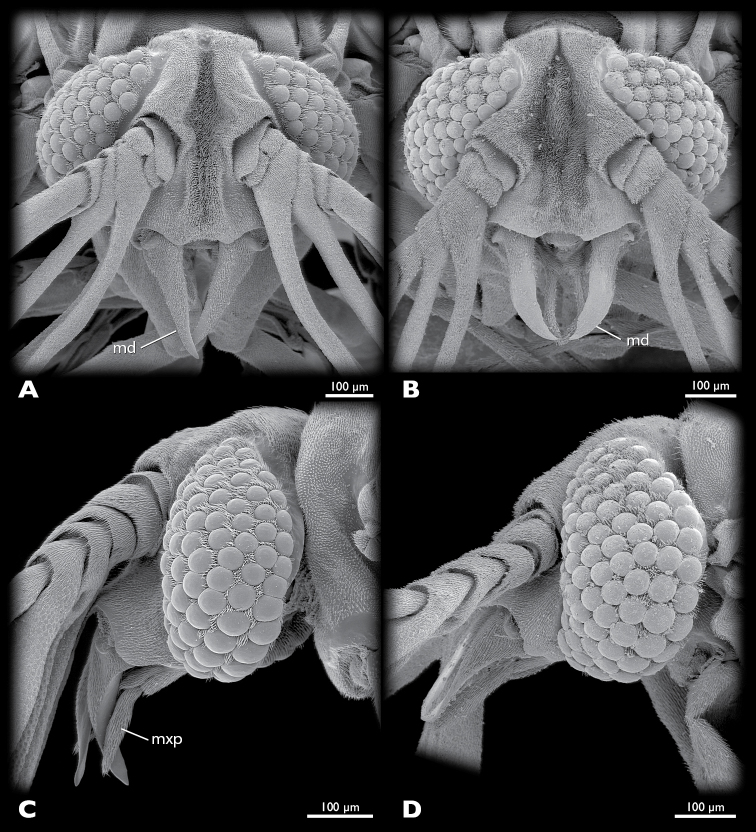
Heads of males of *Mengenilla moldrzyki* sp. n. **A, C** and *Mengenilla chobauti* from Sicily **B**, **D**; **A, B** Frontal view; **C, D**: lateral view; SEM micrographs.

**Figure 4.**
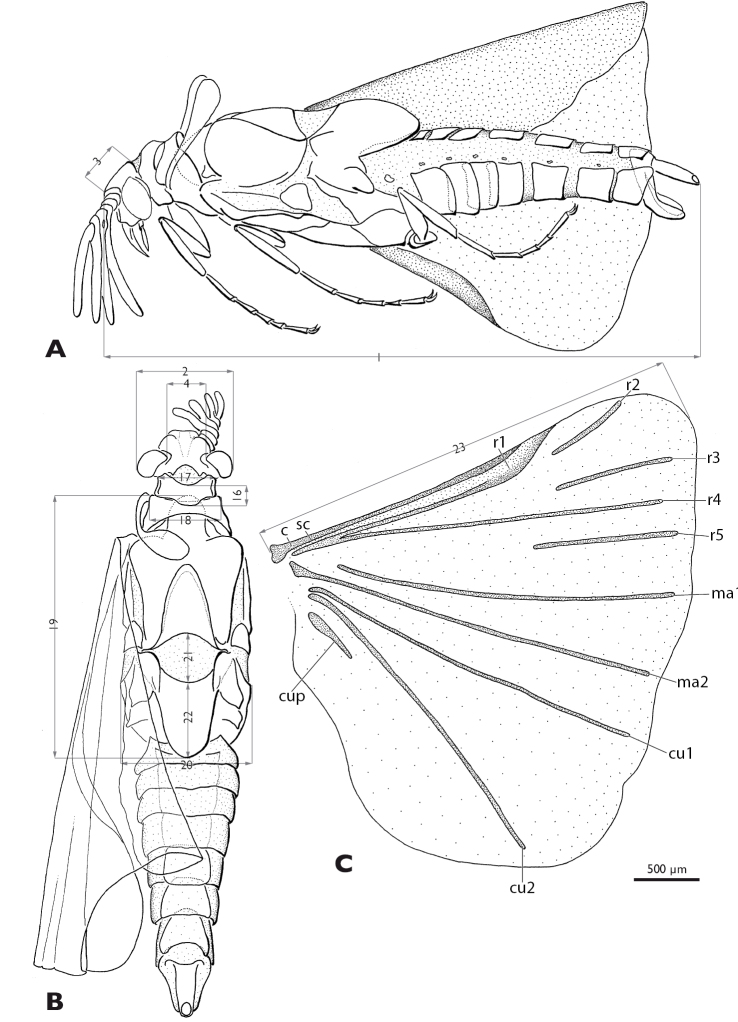
*Mengenilla moldrzyki* sp. n. ♂ with definitions of the measurements **A** Lateral view **B** Dorsal view **C** Hind wing; measurement lines grey. 1: total length, 2 width of the head, 3 length of head, 4 width between compound eyes, 16 length of pronotum, 17 width of pronotum, 18 width of mesonotum, 19 length of metanotum, 20 width of metathorax, 21 length of postlumbium, 22 length of metapostscutellum, 23 length of hind wing; c, costa; cu1, cu2, cubitus anterior; cup, cubitus posterior; ma1, ma2, media anterior; r1–r5, radius; sc, subcosta

**Figure 5.**
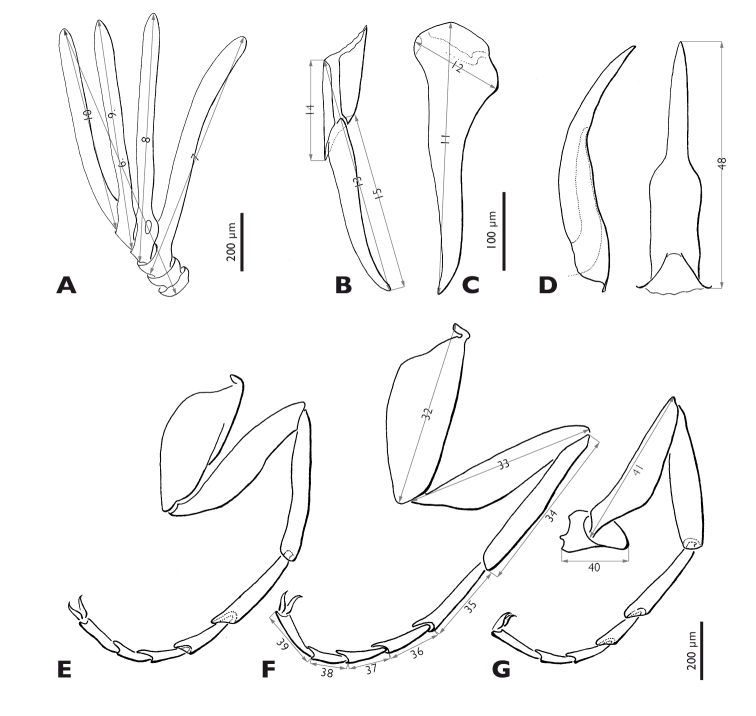
*Mengenilla moldrzyki* sp. n. ♂ with definitions of the measurements **A** Antenna, caudal view **B** Left maxilla, lateral view **C** Right mandible, medio caudal view **D** Penis, lateral and dorso caudal view **E–G** Pro-, meso-, metathoracic leg, ventral view; 6 total length of antenna, 7 length of flabellum antennomere three, 8 length of flabellum of antennomere four, 9 length of flabellum antennomere five, 10 length of antennomere six, 11 length of mandible, 12 width of mandible, 13 total length of maxilla including palp, 14 length of proximal part of maxilla (cardo+stipes), 15 length of maxillary palp, 32 length of mesocoxa, 33 length of mesotrochanterofemur, 34 length of mesotibia, 35–39 length of mesotarsi (proximal to distal), 40 length of metatrochanter, 41 length of metafemur, 48 length of penis.

**Figure 6.**
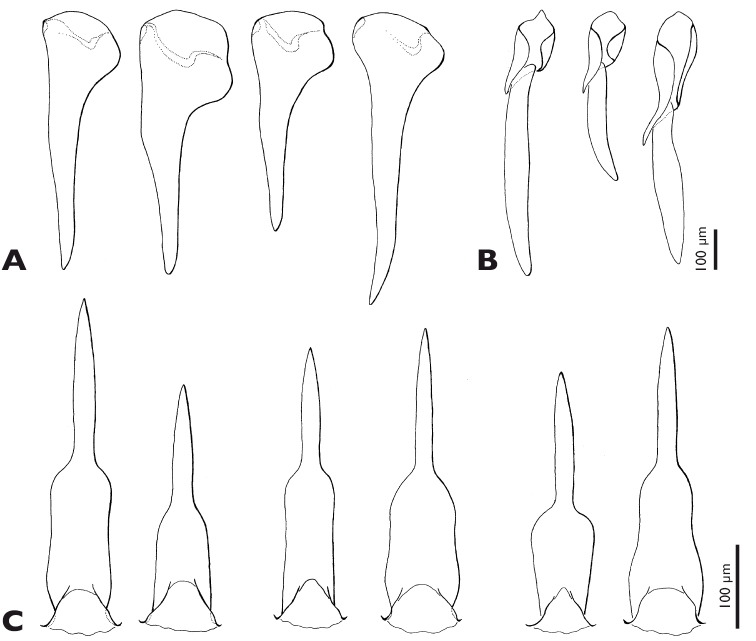
*Mengenilla moldrzyki* sp. n. ♂, variability of mandible, maxilla, and penis **A** Mandibles, medio caudal view **B** Maxilla, lateral view **C** penis, dorso caudal view.

Head capsule (Figs 2, 3A, 3C): dorsal side cream-coloured, lateral part brownish, subgenal region distinctly darkened at mandibular base and postoccipital ridge. Subprognathous, slightly to distinctly inclined; broader than long and strongly narrowed immediately posterior to compound eyes, not retracted into prothorax; laterocervicalia absent; posterodorsal margin of head emarginated; dorsal side densely covered with microtrichia; some short setae present on vertex, but absent from frons; area posterior to compound eyes and ventral side of head capsule glabrous and very smooth; ocelli absent; compound eye very large and extending to ventral side of head, composed of 44–77 large ommatidia (avg. 65); mediodorsal part creamy-white, lateral and ventral part seal brown; dorsal and lateral ommatida widely separated and intervals densely covered with microtrichia; ventral ommatidia slightly larger and closely adjacent; deep lyriform frontal impression present on dorsal side of head and longitudinal bulge laterally; bulges camel-coloured, anteriorly forming antennal insertion; transverse frontoclypeal strengthening ridge absent; anterior clypeofrons slightly emarginated, laterally separated from genal region by dark narrow zone ending posteriorly at antennal insertion; median part parallel-sided, brighter and more densely covered with microtrichia than lateral region; cranial part inflected, separated from frontal mandibular base and mouthfield sclerite by membranous area with distinct median brownish stripe extending laterad; subgenal area and gena seal brown; ventral head closed by lateral postgenal area and median undivided labial plate between maxilla and postgenal region; mouth opening transversely oval.

Antenna (Figs 2A, 5A): large, inserted at anterior end of dorsal longitudinal bulge; scapus and pedicellus very short, broad and cup-shaped, slightly conical at base; flabella of antennomeres 3–5 long, flattened, with rounded tips, decreasing in length from proximal to distal antennomeres, bole-coloured; antennomere 6 distinctly shorter than flabellum of antenommere 5; orifice of Hofeneder’s organ on ventrobasal part of antennomere 5 oval; flagellomeres and flabella densely covered with dome-shaped chemoreceptors.

Labrum: absent.

Mandible (Figs 2A, 3A, 5C, 6A): elongate, slender, and stylet-like; basal part rather narrow and triangular in cross section, seal brown; distal part slightly curved, medially intercrossing in resting position, translucent, brown; lateral and frontal side densely covered with microtrichia except for apical region; ventral side largely glabrous.

Maxilla (Figs 3C, 5B, 6B): basally fused with ventral wall of head capsule without articulatory membrane; surface covered with microtrichia; palp slightly curved inwards, apically attached on proximal element of maxilla (cardo+stipes), densely covered with microtrichia, without sensory spot.

Thorax (Figs 2B, 4B): pronotum v-shaped, with concave anterior margin and rounded caudal margin; slightly overlapping with anterior rim of mesonotum mesally; mesonotum broader than pronotum, nearly as broad as head, with concave caudal rim; prescutum forming distinct bulge, anteriorly extended; metascutum anteriorly with large lobes, covered with sensilla; metascutellum triangular; postlumbium distinctly wider than long, slightly convex anteriorly, strongly convex posteriorly, beige, translucent; metapostnotum slightly longer than wide; posterior margin rounded.

Legs (Figs 5E–F): slender, basitarsus of all legs longer than other tarsomeres; tarsomeres 2–4 decreasing in length; distitarsus of all legs almost as long as tarsomere 2, with two well developed claws; trochanter of hind leg ear-shaped.

Halteres (Fig. 2B): slender, slightly longer as mesonotal width.

Hind wing (Fig. 4C): with typical venation of the genus; veins R2, R3, R5, MA1, Cu1, Cu2, and CuP detached; MA1, MA2, Cu1, and Cu2 reaching almost wing margin; colour beige, veins camel-coloured.

Abdomen (Figs 4, 5D, 6C): tergites less strongly sclerotised than sternites, brown; tergite and sternite of segment I reduced; tergite II partly covered by metapostnotum; tergites II–VIII, rectangular, increasing in width from segments II–VIII; pleural membrane of segments I–VIII wide, camel coloured; spiracles present on segments I–VII; shape of sternites II–VIII similar to corresponding tergites but distinctly broader, shovel-shaped, brown; segment IX strongly sclerotised, distinctly narrower than segment VIII, with caudally elongated subgenital plate; segment X tube-like, extending above tip of subgenital plate; penis curved, with bulbous proximal part; acumen thin, tapering towards apex.

#### Description of the female (Figs 7–10).

Measurements: total length 3,200, width of head 590, width between compound eyes 400, 11–12 ommatidia, total length of antenna 270, length of scapus 20, length of pedicellus 30, length of 3^rd^ antennomer 80, length of 4^th^ antennomer 140, length of mandible 200, width of mandible 90, length of maxilla 400, length of maxillary palp 70, length of procoxa 240, length of protrochanterofemur 290, length of protibia 200, length of protarsi 90, 80, 130 (proximal to distal), length of mesocoxa 270, length of mesotrochanterofemur 330, length of meso- tibia 200, length of mesotarsi 100, 100, 150 (proximal to distal), length of metacoxa 330, length of metatrochanterofemur 400, length of metatibia 230, length of metatarsi 110, 90, 150 (proximal to distal).

**Figure 7.**
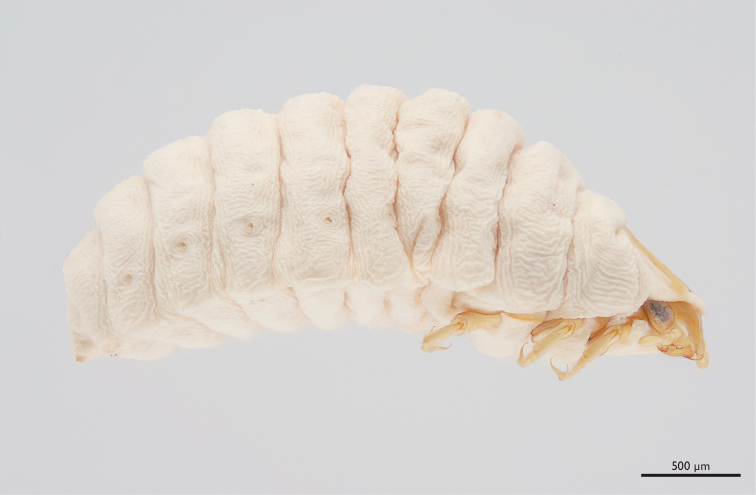
*Mengenilla moldrzyki* sp. n. ♀, lateral view; photomicrograph.

**Figure 8.**
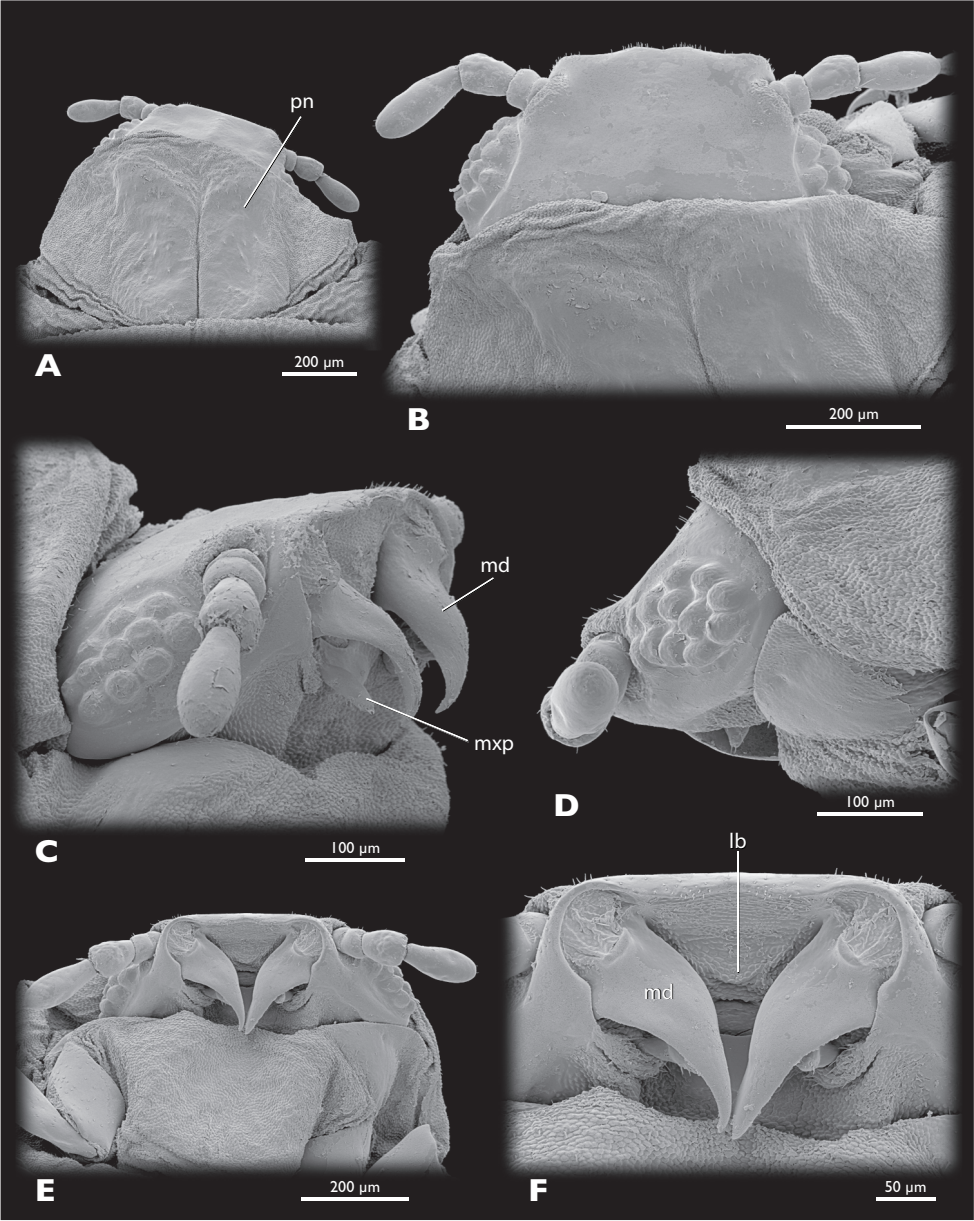
*Mengenilla moldrzyki* sp. n. ♀ **A** Head and prothorax, dorsal view **B** Head, dorsal view **C** Head oblique lateral view **D** lateral view **E** Head and prothorax, ventral view **F** Mouthparts, ventral view; lb, labrum; md, mandible; mxp, maxillary palp; pn, pronotum; SEM micrographs.

**Figure 9.**
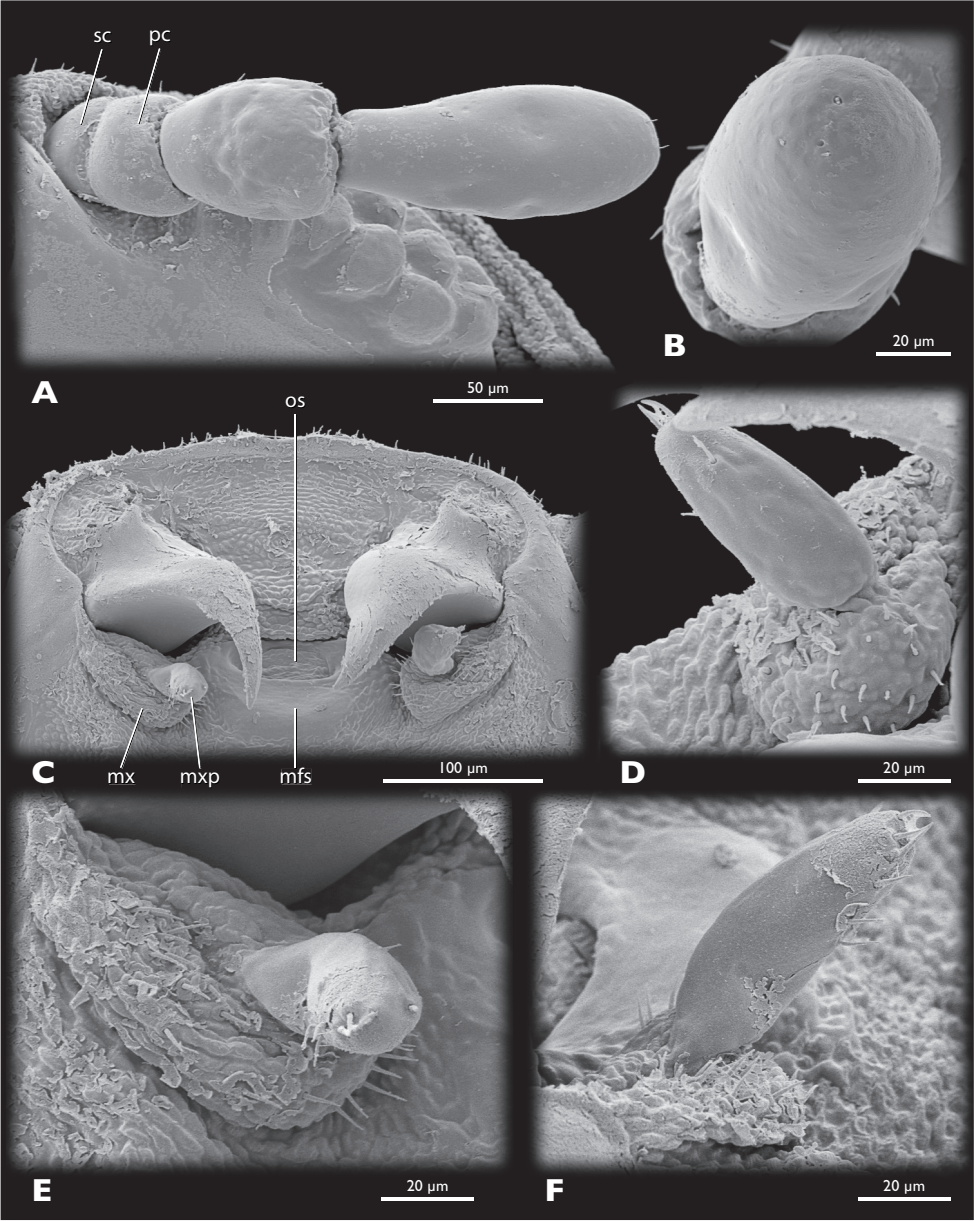
*Mengenilla moldrzyki* sp. n. ♀, details of head **A** left antenna, ventral view **B** Sensilla placodea on antennomere 4, lateral view **C** Mouthparts, ventral view **D** Left maxilla, mesal view **E** Left maxilla, ventral view **F** Left maxilla, lateral view; mfs, mouthfied sclerite; mx, maxilla; mxp, maxillary palp; os, mouth opening; pc, pedicellus; sc, scapus; SEM micrographs.

**Figure 10.**
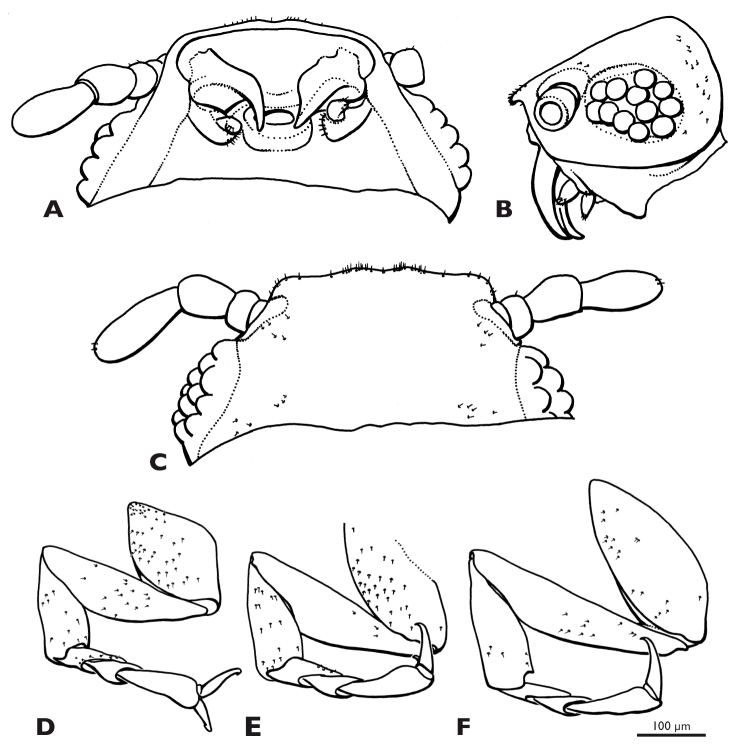
*Mengenilla moldrzyki* sp. n. ♀; **A–C** Head, ventral, lateral, dorsal view **D–F** Pro-, meso-, metathoracic leg, ventral view.

Head capsule (Figs 7–9, 10A–10C): uniformly tan, with the exception of articulatory membranes of antenna and membranised ventral head areas. Relatively small, distinctly broader than long, and cuneiform in lateral aspect; orthognathous, with the posterior part distinctly retracted into the prothorax; laterocervicalia absent; dorsal side smooth, without vestiture of microtrichia; short setae present on anterior clypeal region, few setae present above antennal insertion and area posterior to compound eyes; ocelli absent; compound eye small, composed of 11–12 large, equally sized and closely adjacent ommatidia; microtrichia between ommatidia absent; central part of compound eye grey, peripheral ommatidia tan; circumocular ridge slightly darker; frontal impression on dorsal side of head absent; ventral side of head closed by membranised reduced labium and possibly cervical membrane; mouthfied sclerite small, oval, reaching posterior margin of maxilla; mouth opening transverse, oval.

Antenna (Fig. 9A): small, with 4 segments, without flabella; articulating with broad antennal membrane at anterior end of compound eye; scapus very short, conical, broadly connected with globular pedicellus; antennomere 3 bell-shaped, as long as scapus and pedicellus combined; antennomere 4 club-shaped, as long as antennomeres 1–3; few short setae inserted on distal margin of pedicellus and antennomere 4; Hofeneder’s organ absent, flagellomeres densely covered with sensilla placodea.

Labrum (Fig. 8F): very small fold anterior to mouth opening; setae absent.

Mandible (Figs 8C, F, 9C, 10A, B): hook-shaped; axis of articulation oblique, almost horizontal; secondary joint reduced; basal part broad and triangular in cross section; anterior rim convex, posterior border concave, apical part very slender, not intercrossing in resting position.

Maxilla (Fig. 9C–9F): pear-shaped, weakly sclerotised; insertion adjacent to primary mandibular joint; apex with ~20 stout setae; palp pin-shaped, slightly curved outwards, attached subterminally, with ~10 stout setae distally; sensory spot absent.

Thorax (Fig. 7): ivory-coloured and very weakly sclerotised, with the exception of tan pronotum and pleural sclerites; prothorax trapezoid in dorsal view, with rounded anterior and posterior border; anterior border of pronotum v-shaped, posterior border convex; pronotum divided into two longitudinal plates with broad, weakly sclerotised mesal area, each with ~35 short setae in anterior third; caudal region with about 5–6 short setae; meso- and metathorax dorsally strongly arched; nota of meso- and metathorax not present as sclerotised elements.

Legs (Fig. 10D–10F): stout, inserted laterally; tarsi with 3 segments; distitarsus of all legs almost as long as tarsomeres 1+2 combined; distitarsus with well developed claws.

Abdomen (Fig. 7): ivory-coloured and very weakly sclerotised; abdominal segments strongly arched dorsally, ± flattened ventrally; spiracles at lower third of segments I–VII; fissure-shaped birth opening present on posterior border of segment VII.

COI sequence: 100% identical between the female and the sequenced males.

#### Description of the female puparium (Fig. 11).

Measurements: total length 5,700, maximum width 2,800, maximum height 1,600, length of legs without claws 300.

Pro- and mesothorax fulvous, metathorax and abdomen reddish-brown, lateral side of abdomen with clearly separated fulvous stripe; cuticle shiny, cuticular thorns absent.

Head missing (already shed); dorsal side of puparium strongly arched, ventral side flattened; anterior margin of prothorax rounded and forming distinct bulge with rounded anterolateral edge; anterior third of metathorax slightly constricted; legs very short, inserted laterally, with thread-like claws; caudal margin of abdomen tapering; spiracles present at abdominal segments I–VII.

**Figure 11.**
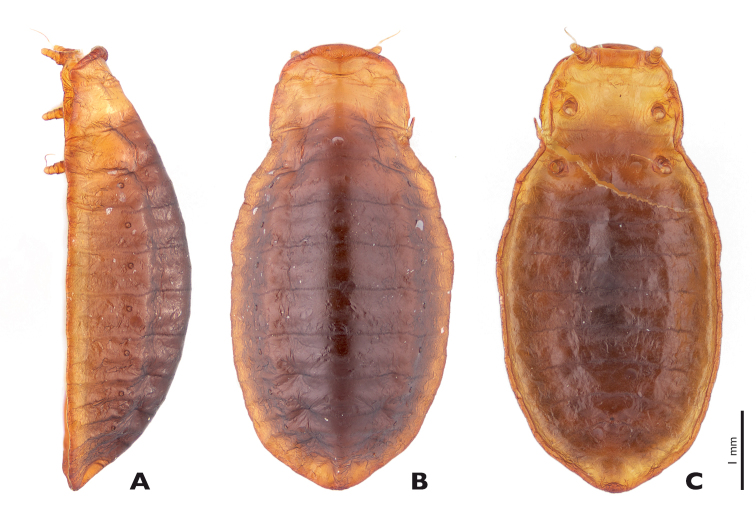
*Mengenilla moldrzyki* sp. n. ♀ puparium **A** lateral **B** Dorsal **C** Ventral view; photomicrographs.

#### First instar larva.

unknown

#### Host.

unknown

#### Material examined.

Tunisia: Grand Erg Oriental, Parc Nationale de Jebil, 265 ♂♂, 33°00'05"N, 9°01'13"E, 11.–15.x.1999, black light, leg. H. Pohl (1 ♂ holotype, on slide, PMJ, 33 ♂♂ paratypes, on slide, PMJ; 40 ♂♂ paratypes, on slides, SDEI; 159 ♂♂ paratypes, in ethanol, HP); same locality, 1 ♀ paratype, prepared from puparium, 08.xi.2005, excavated from sand, leg. U. Moldrzyk, HP, SEM-preparation; same locality, date and collector, 1 ♀ paratype, puparium, HP, dry preparation. Excluded from the type series: 15 ♂♂ (DNA extraction for genome sequencing; Niehuis et al. in press), 10 ♂♂ (poor preserved, SEM, histology).

Type locality and distribution: Parc Nationale de Jebil and surroundings (33°00'05"N, 9° 01'13"E), Tunisia (Fig. 13).

**Figure 12.**
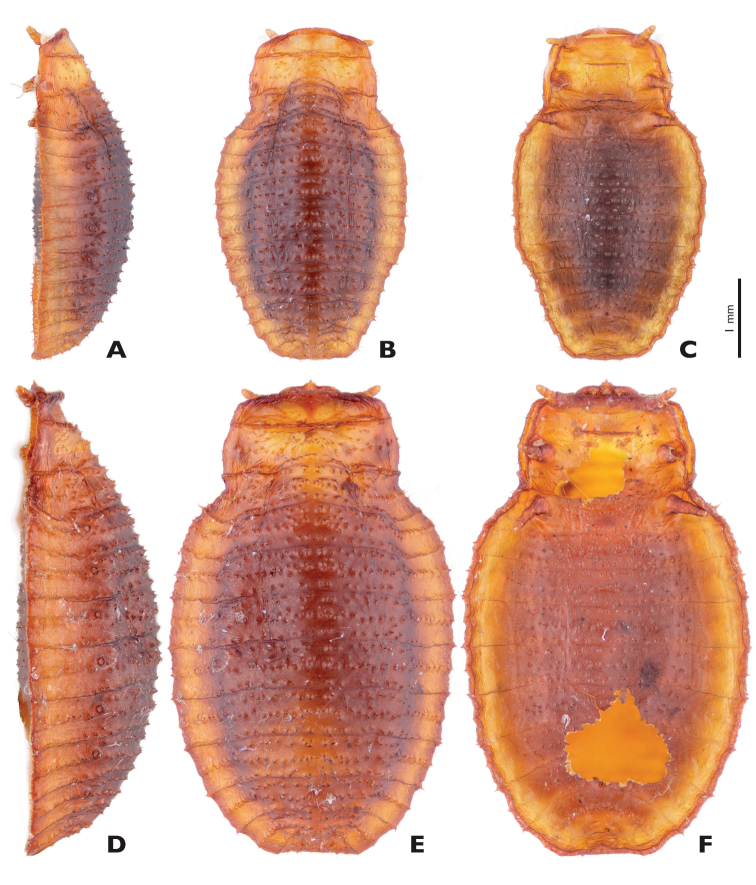
*Mengenilla chobauti* (Sicily) variations of ♀ puparia; **A, D** lateral; **B, E** Dorsal; **C, F** Ventral view; photomicrographs.

**Figure 13.**
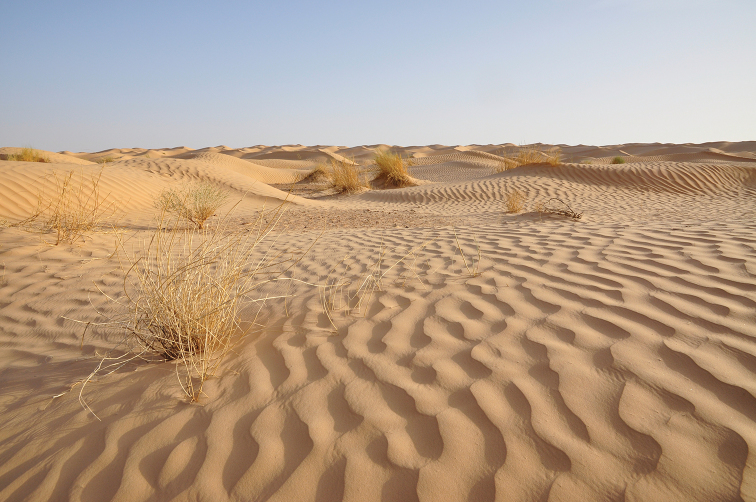
Type locality of *Mengenilla moldrzyki* sp. n. Parc Nationale de Jebil, Grand Erg Oriental, Tunisia.

#### Compared material.

*Mengenilla chobauti* (Figs 3B, 3D, 12), Italy, Sicily, Randazzo, 37°52'17"N, 14°57'02" E, 06.–10.viii.1994, leg. H. Pohl, 5 ♂♂ reared from puparia, HP, in ethanol and SEM-preparation; 20 ♀ puparia, HP, dry preparation.

*Mengenilla santchii* (Pierce, 1918), Tunisia, Kairouan, viii.1907, F. Santchi, holotype ♂, on slide. Original labels: “*Mengenilla chobauti*; HOFENEDER (*Tetrozocera santchii* PIERCE); [Holotype]; Kartei-Nr. 0360; R. KINZELBACH; det. 1970” [handwritten and printed label]; “Kairouan, Marokko; VIII.1907, ♂; ex:?; USNM No. 21434; leg. F. Santchi” [handwritten and printed label]. NMNH.

*Mengenilla theryi* (Hofeneder, 1926), Morocco, Rabat, 17.vii.1923, A. Théry, holotype ♂, on slide. Original labels: “*Mengenillopsis theryi* Hfnd., Rabat. Maroc.; Type. B.M. 1930.193.” [red printed label], “*Mengenillopsis theryi* Hfnd. ♂; Rabat, Maroc.; volant à la lumiere artificielle.; 17 juillet 1923; Voez: Bull. Soc. Sc. Nat. Maroc; VI (1926) p. 56–64. Fig 1–10.; Type; leg. Dr. A. Thèry” [handwritten label]; NHM.

*Mengenilla mauretanica* (Hofeneder, 1928), Morocco, Rabat, vii.1923, leg. A. Théry, 1 ♂, embedded laterally on slide. Original labels: “Théry N° 2” [handwritten label], “*Mengenillopsis mauritanica* Hfnd.; Rabat, Maroc. (a. Théry); B.M.1930.193” [handwritten label], “*Mengenillopsis mauretanica*,♂; Rabat, Maroc.; Volant à la lumière artificielle; leg. Dr. A. Thèry – juilett 1923.; Voyes: Bull. Soc. Sc. Nat. Maroc VIII. (1928) p. 195–211. Fig. 4, 5, 6” [handwritten label] “L`exemplaire N° 2 dans la preparation en situation inverse compare avec les dessins fig. 5 et. 6.” [handwritten label]. NHM. – Same locality, leg. A. Théry, 1 ♂, embedded dorsally on slide. Original labels: “Théry N° 3” [handwritten label], “*Mengenillopsis mauritanica* Hfnd.; Rabat, Maroc. (a. Théry); B.M.1930.193” [handwritten label]. NHM. – Same locality, leg. A. Théry, 1 ♂, embedded laterally on slide. Original labels: “Théry N° 5a” [handwritten label], “*Mengenillopsis mauritanica* Hfnd.; Rabat, Maroc. (a. Théry); B.M. 1930.193” [handwritten label]. NHM. – Same locality, leg. A. Théry, 1 ♂, on slide. Original labels: “*Mengenillopsis mauritanica* Hfnd.; Rabat, Maroc. (a. Théry); B.M. 1930.193” [handwritten label], “Rabat (Maroc); A. Théry leg.; R.Ph.D. prepar.” [handwritten label], “*Mengenillopsis mauretanica* ♂; Rabat, Maroc.; volant à la lumière artificielle; leg. Dr. A. Théry … aout 1923.; Voyes: Bull. Soc. Sc. Nat. Maroc; VIII. (1928) p. 195–211. Fig. 1.” [handwritten label]. NHM. – Same locality, leg. A. Théry, 1 ♂, on slide. Original labels: “*Mengenillopsis mauritanica* Hfnd.; Rabat, Maroc. (a. Théry); B.M. 1930.193” [handwritten label], “Rabat (Maroc); A. Théry leg.; R.Ph.D. prepar.” [handwritten label], “*Mengenillopsis mauretanica* ♂; Rabat, Maroc.; volant à la lumière artificielle; leg. Dr. A. Théry … aout 1923.; Voyes: Bull. Soc. Sc. Nat. Maroc; VIII. (1928) p. 195–211. Fig. 2, 7.” [handwritten label]. NHM.

*Mengenilla* cf. *chobauti*, Tunisia, Bou Hedma National Parc, 34° 29’ 40” N; 09° 38’ 37” E, 06.x.1999, leg. H. Pohl. 2 ♀♀ puparia, HP, dry preparation.

*Mengenilla gracilipes* (Lea, 1910), Australia, Bridgetown, Western Australia, at light, Syntype ♂, on slide. Original labels: “*Mengenilla gracilipes* (Lea, 1910); Cotype, B.M. 1910.323; Bridgetown W.A.; to lights; leg. Lea; det. Kinzelbach 1968” [handwritten label]. “Bridgetown; W.a. (Lea); To lights” [handwritten label, probably original label of Lea]; NHM.

*Mengenilla orientalis* Kifune and Hirashima, 1980, Sri Lanka, Mannar District, 10 miles NW of Mannar, 04.–05.xi.1976, black light, leg. G.F. Hevel, R.E. Dietz IV, S. Karunaratne, D.W. Balasooriya, holotype ♂, in ethanol. Original labels: “Holotype, *Mengenilla orientalis*, Kifune et Hirashima” [handwritten label]. “SRI LANKA: Man. Dist.; Olaithoduvae, 10 mi NW; of Mannr, 0–50 feet; black light; 4–5 November 1976” [printed label]. “Collected by: G.F. Hevel, R.E. Dietz IV, S. Karunaratne; D.W. Balasooriya” [printed label]. “USNM #; 76712” [printed label]; NMNH.

*Mengenilla australiensis* Kifune and Hirashima, 1983, Australia, Northern Territory, 8 km N of Kulgera, 03.iv.1980, blacklight, leg. G.F. Hevel, J.A. Fortin, paratype ♂, in ethanol. Original labels: “*Mengenilla australiensis*; PARATYPE” [handwritten label], “AUSTRALIA: N.T.; 8 KmN of Kulgera; at blacklight; 3 April 1980; GFHevel & JAFortin” [printed label]; NMNH.

*Mengenilla kaszabi* Kinzelbach, 1970, Mongolia, Bajanchongor Aimak, Oasis Echin gol, about 90 km NE borderguard Caganbulag, 950 m, 28.vi.1967, at light, leg. S. Kaszab, paratype ♂, on slide. Original labels “*Mengenilla kaszabi* Kinzelbach ♂ Paratypus, Kartei-Nr. 0128, R. KINZELBACH det. 1969“ [handwritten and printed label], “Mongolia, Bajanchongor aimak: Oase Echin gol, 90 km von Caganbulag, 28.VI.1967, an Licht, leg. S. KASZAB” [handwritten label]; HP.

*Mengenilla arabica* Kinzelbach, 1979, Kuwait, 04.vi.1983, leg. W. al-Houty, ♂, on slide. Original labels “*Mengenilla arabica* KINZELBACH, 1979, Kartei-Nr. 1217, R. Kinzelbach det. 1984” [handwritten and printed label], “W Kuwait, 4.06.1983, ex: …, det. …, leg. W. al-Houty” [handwritten and printed label]; HP.

#### Distribution.

*Mengenilla moldrzyki* sp. n. is only known from the type locality.

#### Ecology and phenology.

In contrast to *Mengenilla chobauti*, *Mengenilla moldrzyki* sp. n. apparently only occurs in pure sand dune areas. Despite intense search no puparia were found among widely scattered stones in the habitat during collecting trips in 1999 and 2010. The puparia might be buried in sand, as suggested by the discovery of a female within its puparium in such a situation. The reduction of the characteristic cuticular thorns of the puparium is likely related to this lifestyle. The thorns function as attachment structures that are frequently additionally enhanced by using silk spun from spiders under rocks and stones (Pohl and Beutel 2008).

Presently, no statements can be made on the seasonal occurrence of the adult males of *Mengenilla moldrzyki* sp. n. before and beyond October. However, it was observed that the period of activity (and life span) of the adult males is very short (maximum ca. 2 hours). In October 1999 and 2010, males were only found about half an hour after sunset (~6.30 p.m.). No specimens were found after 8.30 p.m. Most of the flying males were observed between 6.30 p.m. and 8.00 p.m. Captured males lost their ability to fly approximately 2 hours after they had been captured and died half an our later.

## Discussion

The type locality of *Mengenilla chobauti* is Ain Sefra in Algeria. All other species of *Mengenilla* described from northern Africa (i.e., *Mengenilla theryi* (Hofeneder, 1926), *Mengenilla mauretanica* (Hofeneder, 1928) (both from Morocco), *Mengenilla santchii* (Pierce, 1918) (from Tunisia)) are today considered as junior subjective synonyms of *Mengenilla chobauti* (Kinzelbach 1970, Cook 2007). We re-examined all available material (including all types) of these species, and encountered a considerable amount of variation, especially in the length of antennomere 6, the length of the flabella, and the shape of the maxilla and penis. As pointed out by Cook (2007), it cannot not be excluded that more than one species occurs in this region. However, all synonymised North African species, including *Mengenilla santchii* from Tunisia, differ significantly from *Mengenilla moldrzyki* by the broad distal part of their mandible.

### Recommendations for future species descriptions

Important diagnostic features of males of species of the genus *Mengenilla* are the shape of the posterodorsal margin of head, the dorsomedian frontal impression, the total length of the antennae, the tips of the flabella and antennomere 6, the shape of the distal part of the mandibles, the proximal part of the maxilla, the insertion of the maxillary palp, the shape of the pronotum, and the length of the metapostscutellum. In contrast, the total length, the proportions of the maxilla and the maxillary palp as well as the shape of the penis are very variable and only partly suitable for species identification (compare Fig. 6). Kinzelbach (1979) and Cook (2007) refer to the different patterns of microtrichia on the mandibles. This feature seems to be suitable for diagnosis, but the vestiture varies greatly on the frontal, lateral and ventral mandibular areas (Fig. 3). It is not clear from previous descriptions and illustrations from which perspective the isolated mandibles are shown. This greatly reduces the value of the presented information (see Cook 2007: Fig 5). If more than one specimen is available, standard views of the head in dorsal, frontal, and lateral view should be given (and documented by SEM if possible) in addition to drawings of the antenna, mandible, maxilla, dorsal view of the whole insect, the legs, the hind wing, and the penis. With the use of the SEM specimen holder developed by Pohl (2010), a single specimen can be examined from all sides.

Differences in colouration also appear suitable for diagnosis but have rarely been used in the literature so far. Unfortunately, older specimens treated with potassium hydroxide solution and embedded in Canada balsam on slides are no longer useful for this purpose. To document the colouration, light micrographs of the head and thorax in dorsal view and of the head in frontal view should be given. Only with well-documented descriptions, a reliable identification of other conspecific individuals is possible without comparing it to the type specimens.

## Supplementary Material

XML Treatment for Mengenilla moldrzyki
